# A single-cell RNA sequencing atlas of the healthy canine lung: a foundation for comparative studies

**DOI:** 10.3389/fimmu.2025.1501603

**Published:** 2025-03-06

**Authors:** Elodie Rizzoli, Laurence Fievez, Aline Fastrès, Elodie Roels, Thomas Marichal, Cécile Clercx

**Affiliations:** ^1^ Department of Companion Animals Clinical Sciences, Faculty of Veterinary Medicine, University of Liège, Liège, Belgium; ^2^ Department of Functional Sciences, Faculty of Veterinary Medicine, University of Liège, Liège, Belgium; ^3^ Laboratory of Cellular and Molecular Immunology, GIGA Institute, University of Liège, Liège, Belgium; ^4^ Laboratory of Immunophysiology, GIGA Institute, University of Liège, Liège, Belgium; ^5^ Walloon Excellence in Life Sciences and Biotechnology (WELBIO) Department, WEL Research Institute, Wavre, Belgium

**Keywords:** dog, lung, healthy, single-cell RNA sequencing, pulmonary cells, gene expression, markers

## Abstract

Single cell RNA sequencing (scRNA-seq) can be used to resolve the cellular and molecular heterogeneity within a tissue by identifying cell populations with an unprecedented granularity along with their transcriptional signatures. Yet, the single cell gene expression profiles of cell populations in the healthy canine lung tissue remain unexplored and such analysis could reveal novel cell populations or markers lacking in dogs and facilitate comparisons with lung diseases. Using fresh healthy lung biopsies from four dogs, we conducted droplet-based scRNA-seq on 26,278 cells. We characterized 46 transcriptionally distinct cell subpopulations across all lung tissue compartments including 23 immune, 13 mesenchymal, five epithelial and five endothelial cell subpopulations. Of note, we captured rare cells such as unconventional T cells or Schwann cells. Differential gene expression profiles identified specific markers across all cell subpopulations. Fibroblasts clusters exhibited a marked transcriptional heterogeneity, some of which might exert immune regulatory functions. Finally, the integration of canine lung cells with an annotated human lung atlas highlighted many similarities in gene expression profiles between species. This study thus provides an extensive molecular cell atlas of the healthy canine lung, expanding our knowledge of lung cell diversity in dogs, and providing the molecular foundation for investigating lung cell identities and functions in canine lung diseases. Besides, the occurrence of spontaneous lung diseases in pet dogs, with phenotypes closely resembling those in humans, may provide a relevant model for advancing research into human lung diseases.

## 1 Introduction

Single-cell mRNA sequencing (scRNA-seq) enables high throughput and high-resolution transcriptomic analysis of the heterogeneity of cells within a population by profiling the transcriptome of each cell constituting a biological sample (1). Extensive cell atlases of the human lung have been published and serve as highly valuable references for the analysis of diseased lung (2–4). Although scRNA-seq is still in its premises in non-conventional animal model species, it has already been validated in dogs for the identification and characterization of cellular subpopulations in the bronchoalveolar lavage fluid (BALF) of healthy dogs and dogs affected with canine idiopathic pulmonary fibrosis (1, 5). However, analyzing BALF provides information over only a subset of lung cells and to date, the molecular state of all cells in canine lung tissue has not been investigated yet. A deep understanding of canine lung cell biology is crucial to decipher alterations occurring in parenchymal lung diseases. Such comparisons of cell subpopulations between healthy and diseased dogs should lead to a better understanding of the pathophysiology of lung diseases, which is of interest in the perspective of finding new treatment strategies.

Moreover, the canine species is increasingly recognized as a relevant species to understand human diseases. Indeed, dogs and humans share genetic, anatomical and physiological similarities, similar immune system and immune responses and the same environment and exposures (6–10). The similarities between the human and canine genomes are stronger than between human and mouse for many gene families including those related to cancer for instance (8). Besides lung cancer (11), dogs can spontaneously develop other lung diseases, such as idiopathic pulmonary fibrosis (12, 13) and pulmonary embolism (14), that share features with human conditions, providing thus a model of spontaneously occurring disease. Although pet dogs would never replace experimental mouse models for preclinical mechanistic studies (9), studying the molecular foundations of pulmonary diseases in dogs would provide valuable complementary insights into the pathophysiology of spontaneous diseases.

Accordingly, the aim of the present study was to generate an extensive molecular cell atlas of the healthy canine lung using scRNA-seq and to establish gene expression profiles of all lung cells. Such atlas would provide foundation for investigating disease-related heterogeneity at single cell level.

## 2 Methods

### 2.1 Sample collection

Healthy canine lung tissues were collected either from dogs euthanized for reasons unrelated to this study, or from healthy regions of lung lobes resected for solitary lung tumors, ensuring a margin of at least 2 cm from the visible tumor edge. All dogs were privately-owned. Samples were collected with informed owner consent and under the local Animal Ethics Committee approval (#20-2245). In each dog, one parenchymal lung biopsy was collected directly after death or lobectomy and transported in HBSS (Gibco) containing 5% v/v of FBS (Gibco) on ice for immediate processing. Histopathological evaluation confirmed that the biopsy site was free of lung disease.

### 2.2 Sample preparation

Each lung sample underwent mechanical dissociation with razor blades and was suspended in HBSS + 5% FBS with collagenase A (1 mg/mL; Sigma) and DNase I (0.05 mg/mL; Roche) before incubation at 37°C for 45 min. The cells were then filtered through a 70 µm cell strainer (BD Falcon) and resuspended in PBS (Biowest) containing 10 mM EDTA (Merck Millipore). Red blood cells were lysed as needed with a lysis buffer containing 0.15 M NH_4_Cl, 0.01 M KHCO_3_ and 0.1 mM EDTA at pH 7.5. The final cell suspension contained between 500 and 1000 cells/µL in PBS containing 0.04% of BSA (Sigma) and 0.2 U/µL of RNase inhibitor (Roche). Final cell viability was assessed by trypan blue staining and considered acceptable above 70 percent.

### 2.3 Library preparation and sequencing

Approximatively 10,000 cells from each lung sample were loaded in a Chromium Controller or Chromium iX instrument (10x Genomics, Pleasanton, CA) and encapsuled with unique barcoded primers using the drop-sequencing method according to manufacturer’s instructions. Emulsion breakage, cDNA amplification and libraries construction were performed using Chromium Single Cell 3′ reagent kit v2 (10x Genomics) according to manufacturer’s instructions. Libraries were sequenced with a NextSeq500 system (Illumina, San Diego, CA) with a target of 20,000 reads per cell, which resulted in relatively low saturation (34.1, 55.0, 46.8 and 52.8 percent) but turned out to be sufficient to effectively delineate cell types. Raw sequencing data files (.bcl) were converted to FASTQ format using bcl2fastq v2.20.0.422 (Illumina) and Cell Ranger software version 9.0.0 (10x Genomics) was utilized for aligning sequencing reads in FASTQ files to the dog reference transcriptome (CanFam3.1), filtering, counting unique molecular identifiers, and generating gene-barcode matrices.

### 2.4 Data filtering, integration and clustering

Filtered gene expression matrices were analyzed using Seurat R package version 4.3 (http://satijalab.org/seurat/). Beforehand, each sample was individually processed to eliminate doublets, low-quality or dying cells. Genes expressed in less than 10 cells were excluded, as well as cells expressing less than 200 genes or having more than 20 percent reads assigned to mitochondrial genes. Cell clusters co-expressing distinct canonical markers from two or more tissue compartments were considered as doublets and removed from the datasets. After combining datasets, each sample was normalized with SCTransform, regressing out the effects of the percentage of mitochondrial reads and of the cell cycle score, calculated with the “CellCycleScoring” function. Integration of individual samples was performed using normalized values from SCTransform and the top 3000 variables genes as anchors for canonical correlation analysis. Principal component analysis was used to perform linear dimension reduction and an ElbowPlot was used to determine the number of principal component analysis dimensions to select. Clustering was performed using the Louvain-graph-based algorithm in R and visualized by non-linear dimensional reduction using uniform manifold approximation and projection (UMAP) plots. Ideal clustering resolution was determined using the package clustree (15). The following clustering parameters were used for the integrated dataset: res = 2.8, dims = 100, min.dist = 0.3. Each cluster was assigned to a tissue compartment using their expression of canonical marker genes (*EPCAM* for epithelial, *PTPRC* for immune, *PECAM1* for endothelial cells, the rest being mesenchymal cells). Each compartment was individualized, and integration and clustering was repeated in each subset as described above. The following dimension reduction and clustering parameters were used for final cell subsets; muscle: res = 0.9, dims = 15, min.dist = 0.35; fibroblasts: res = 0.7, dims = 5, min.dist = 0.35; myeloid: res = 1.8, dims = 60, min.dist = 0.35; lymphoid: res = 1.5, dims = 50, min.dist = 0.35; epithelial: res = 0.7, dims = 8, min.dist = 0.35; endothelial: res = 0.8, dims = 12, min.dist = 0.35. Cell cluster identities were determined based on their expression of canonical markers genes described in the literature and their lists of differentially expressed genes (DEGs).

### 2.5 Differential gene expression analysis

The FindAllMarkers function (with Wilcoxon rank sum test adjusted for multiple testing with Bonferroni correction) was used to identify DEGs across clusters. Only DEGs with adjusted P<0.05 were considered. When possible, differential expression analysis was also performed using DESeq2 after pseudobulk conversion (16). Pseudobulk approach was used to compare cell clusters with limited heterogeneity and with at least 15 cells in each sample. DEGs with an adjusted P < 0.05 and a log2(fold change) > 0.58 were considered statistically significant. Using lists of significant positive DEGs, gene ontology (GO) analyses for biological processes were performed with the GO Consortium website (https://geneontology.org/; released on 2024/11/03). GO analyses were performed using Fisher’s Exact test and Bonferroni correction for multiple testing. Statistically significant enrichments were then selected according to their biological relevance.

### 2.6 Data visualization

Clustering was visualized using uniform manifold approximation and projection (UMAP) plots. Gene expression was visualized using feature plots, violin plots and dot plots using SCTransform normalized counts. The absence of cancer cells in lung samples adjacent to a focal tumor was further validated by comparing the expression of growth factor receptor genes (*EGFR* and *ERBB2*) and proliferation marker genes (*PCNA* and *MKI67*) in split feature plots after downsampling the data to obtain equal cell numbers depicted in the feature plot for each condition.

### 2.7 Human lung homology analysis

A fully annotated healthy human lung dataset was obtained from the integrated Human Lung Cell Atlas (HCLA) core, which combines 584 944 healthy lung cells from 107 individuals, re-annotated to generate a consensus cell type reference (4). The lung parenchyma subset of the HCLA core was selected and downsampled to 50,000 cells to facilitate Seurat object management. The gene symbols from the human dataset were converted from human to canine using the convert_orthologs() function from the orthogene package (17). The human and canine datasets were merged, normalized with SCTransform and integrated into one object using the same integration workflow as above. Cell type homologies between species were evaluated using an approach adopted from Ammons et al. (18, 19): The prefix ‘can’ or ‘hu_’ was added to canine and human cell type annotations, and hierarchical clustering was performed using the hclust() function with method set to “complete”.

## 3 Results

### 3.1 Study sample summary

Healthy lung tissue biopsies were collected from four different dogs. Two post-mortem biopsies originated from dogs exempt from lung disease and two originated from the unaffected lung tissue adjacent to a focal primary pulmonary adenocarcinoma. Three biopsies were collected from the periphery of the right caudal lobe and one was collected from the periphery of the right cranial lobe. There were two female and two male dogs; two Pointers, one Cocker and one Beagle crossbreed. They were aged from 5 to 10 years (median 7 years) and weighed from 12 to 30 kg (median 18.8 kg). All samples were confirmed to be free of lung disease by histopathology.

### 3.2 Four lung tissue compartments are individualized

**Figure 1:**
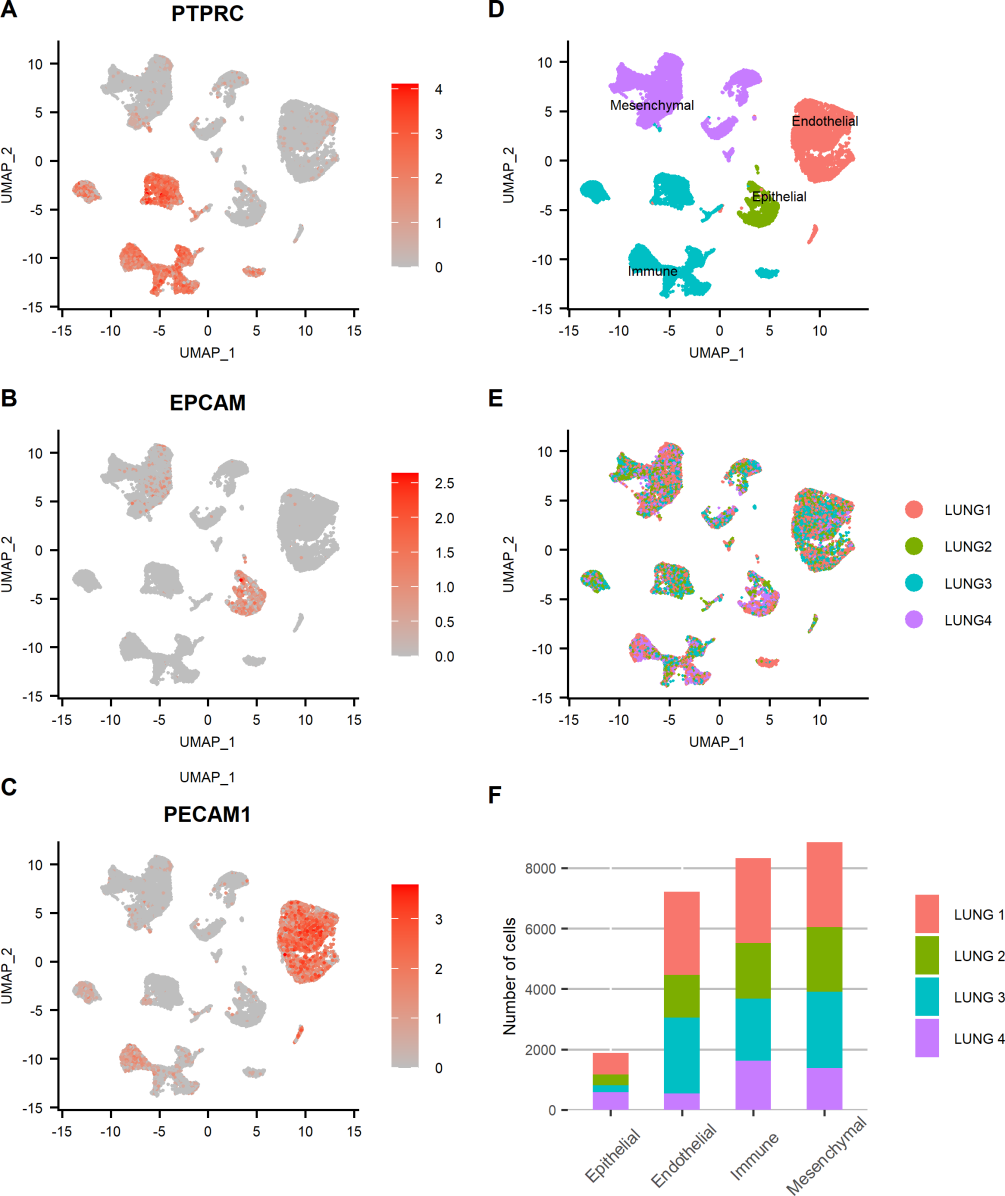
Four major lung tissue compartments were identified and evenly distributed among lung samples. **(A–C)** Feature plots representing the normalized expression of canonical markers used to discriminate lung tissue compartments (*PTPRC*: immune, *EPCAM*: epithelial, *PECAM1*: endothelial, other cells: mesenchymal). Color scales represent the expression level of each gene. UMAP representation of the cells of all lung samples annotated by **(D)** tissue compartment and **(E)** sample origin. **(F)** Bar plot showing the relative contribution of each sample to each tissue compartment.

After tissue dissociation, scRNA-seq was performed on each sample. A total of 26,278 cells sequenced at a depth of 26,900 mean reads per cell passed quality control. A summary of sequencing and mapping quality control metrics for each sample is available in **Table 1**. After integration of samples in Seurat, four major lung tissue compartments were identified thanks to canonical markers expression (**Figures 1A–D**). The expression of *EPCAM* allowed the identification of epithelial cells, *PECAM1* (coding for *CD31*) of endothelial cells, *PTPRC* (coding for *CD45*) of immune cells, and cells expressing neither *EPCAM*, *PECAM1*, nor *PTPRC* were identified as mesenchymal cells (2, 20). Each individual sample contributed to all four tissue compartments without overt batch effect (**Figures 1E, F**). Subsequently, each compartment was individualized and re-clustering was performed within each subset.

**Table 1:** Summary of sequencing and mapping quality control metrics for each sample.

	Lung 1	Lung 2	Lung 3	Lung 4
Number of cells passing quality control	9099	5761	7306	4112
Sequencing saturation, %	34.1	55.0	46.8	52.8
Reads mapped confidently to genome, %	83.6	79.5	81.8	88.1
Reads mapped confidently to transcriptome, %	56.8	48.4	52.2	60.0
Median genes/cell	1.368	873	1,362	2,296
Median unique molecular identifier counts/cell	2,710	1,479	2,880	6,871
Total genes detected	17,455	16,872	17,019	16,835

### 3.3 Lung mesenchymal cells include muscle cells, fibroblasts and Schwann cells

**Figure 2:**
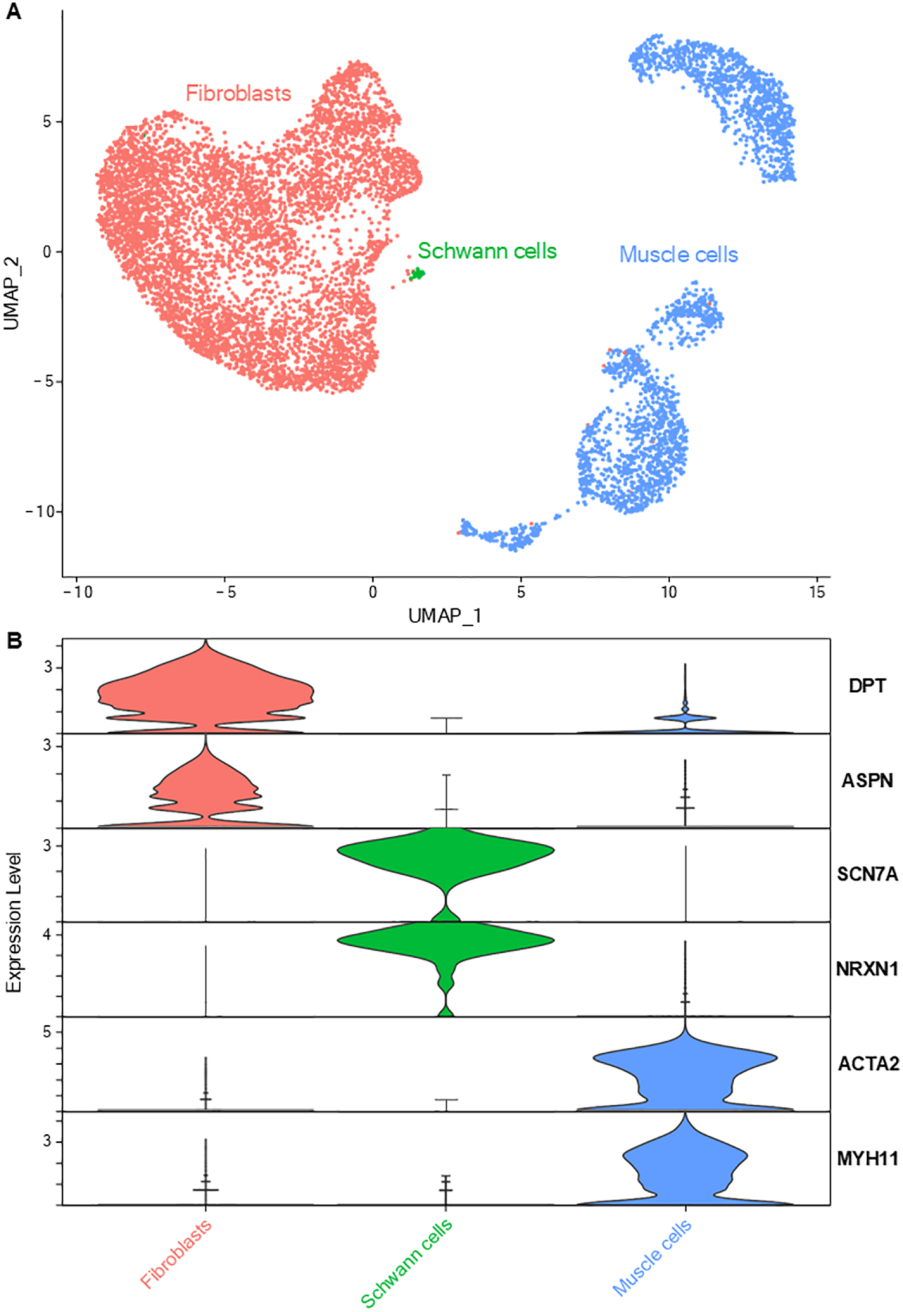
Lung mesenchymal cells. **(A)** UMAP representation of the three main mesenchymal cell types: fibroblasts, Schwann cells and smooth muscle cells. **(B)** Violin plots depicting the specific expression of key genes associated with each main cell type.

The gene expression profiles of mesenchymal cells allowed their characterization into three sub-groups (**Figure 2**): muscle cells (expressing genes of contractility such as *ACTA2*, *TAGLN*, *MYH11*), fibroblasts (overexpressing genes coding for collagens such as *COL1A1* and matrix proteins) and Schwann cells (specifically expressing markers such as *SCN7A*, *NRXN1*, *CDH19* and *NCAM1*) (3, 21, 22). GO analysis based on Schwann cells DEGs revealed an enrichment in ‘axonogenesis’ and ‘myelination’ processes (detailed lists of enriched biological processes from all performed GO analyses are provided in **Supplementary Table 1**).

### 3.4 Lung fibroblast clusters exhibit a marked heterogeneity

**Figure 3:**
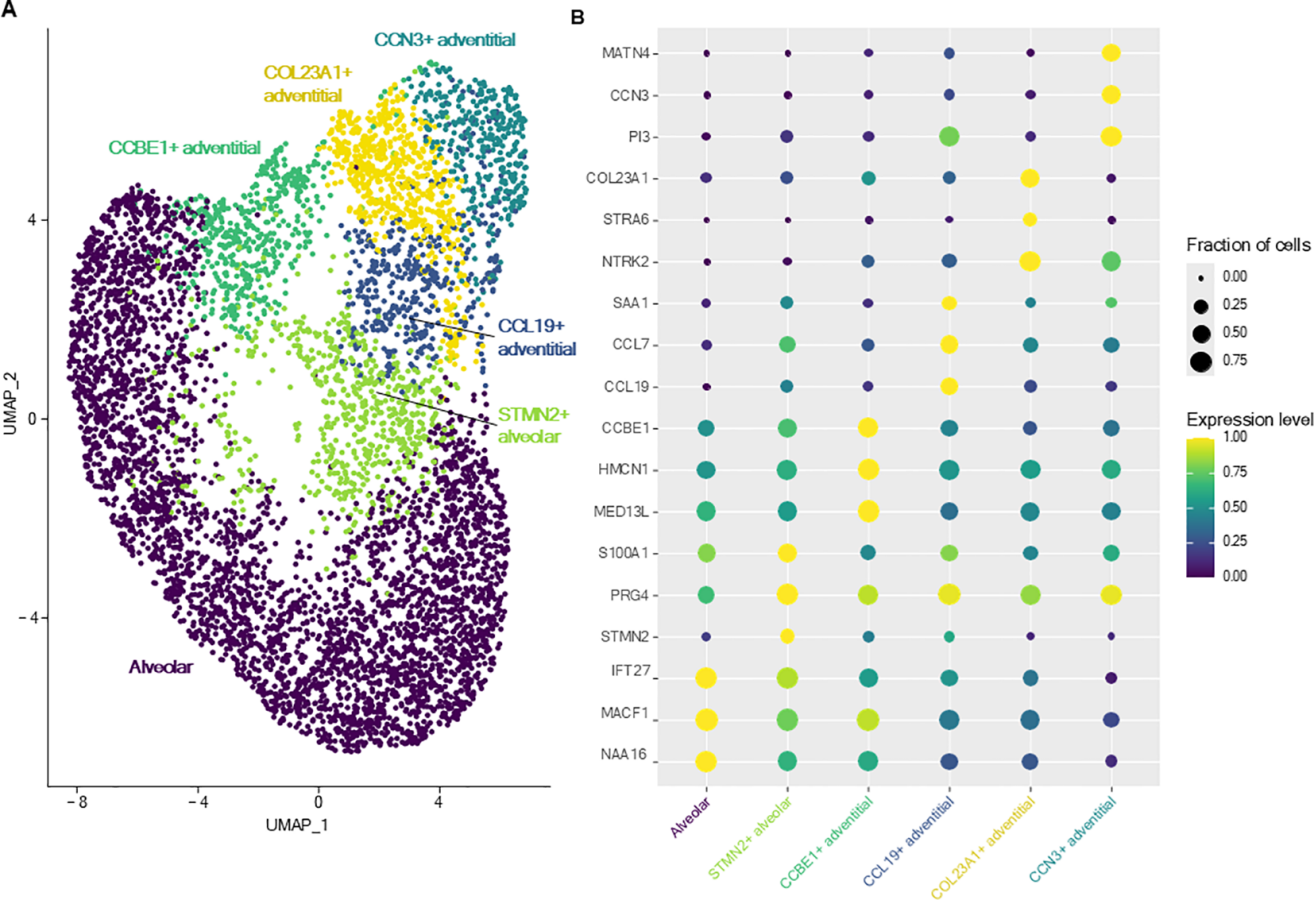
Lung fibroblasts. **(A)** UMAP representation of the six distinct fibroblasts clusters identified in canine healthy lungs. **(B)** Dot plot representing the specific expression of markers by different fibroblasts clusters.

Subclustering and analysis of fibroblasts allowed identification of six transcriptionally distinct clusters (**Figure 3**). The complete list of DEGs of each fibroblast cluster versus all other fibroblasts are provided in **Supplementary Table 2**. In accordance to the 3-axis classification for mesenchymal cells described in the literature (21), fibroblasts were annotated as either adventitial (proximal, located in the bronchovascular bundle) or alveolar (distal) fibroblasts.

Alveolar fibroblasts were annotated based on their overexpression of *COL13A1*, *WNT2*, *NPNT, FGFR4* and *GPC3* (2, 21, 22). One cluster of alveolar fibroblasts, referred to as ‘*STMN2* ^+^ alveolar fibroblasts’ clustered separately from other alveolar fibroblasts. This cluster overexpressed genes such as *STMN2, PRG4, IL33, COL6A6* as well as cytokine and chemokine genes such as *CCL19*, *CXCL12*, *CCL7* and compared with alveolar fibroblasts, GO analysis revealed an enrichment in ‘cytokine-mediated signaling pathway’, ‘inflammatory response’ and ‘positive regulation of cell population proliferation’ (**Table 2**; **Supplementary Table 1**).

**Table 2:** Gene ontology analysis of the transcriptomic profiles of fibroblasts clusters.

Cluster	Biological process	Gene set	Upregulated	Expected	Fold enrichment	P-value
‘*STMN2* ^+^ adventitial fibroblasts’	cytokine-mediated signaling pathway	295	11	1.11	9.9	9.40E-05
	regulation of inflammatory response	235	8	0.89	9.04	1.99E-02
	inflammatory response	304	9	1.15	7.86	1.46E-02
	positive regulation of cell population proliferation	547	12	2.06	5.82	6.30E-03
	regulation of cell migration	626	12	2.36	5.09	2.55E-02
‘*CCL19* ^+^ adventitial fibroblasts’	leukocyte proliferation	86	10	1.18	8.47	1.98E-03
	cytokine-mediated signaling pathway	295	24	4.05	5.92	2.23E-08
	inflammatory response	304	20	4.18	4.79	6.05E-05
	regulation of inflammatory response	235	14	3.23	4.34	3.42E-02
	positive regulation of immune system process	678	33	9.31	3.54	2.22E-06
	regulation of cytokine production	502	23	6.89	3.34	3.55E-03
	immune system process	1516	51	20.82	2.45	1.70E-05
‘*CCN3* ^+^ adventitial fibroblasts’	regulation of cell migration	626	41	15.43	2.66	1.03E-04
	positive regulation of cell population proliferation	547	34	13.49	2.52	6.00E-03
	regulation of transport	950	48	23.42	2.05	1.87E-02
	nervous system development	1414	68	34.86	1.95	7.41E-04
	tissue development	1170	56	28.85	1.94	1.52E-02
	regulation of developmental process	1511	68	37.25	1.83	1.10E-02
‘*COL23A1^+^* adventitial fibroblasts’	positive regulation of cell migration	369	22	5.49	4.01	2.75E-04
	animal organ morphogenesis	671	28	9.98	2.8	6.87E-03
	regulation of cell population proliferation	972	37	14.46	2.56	1.14E-03
‘*CCBE1* ^+^ adventitial fibroblasts’	circulatory system development	612	57	29.13	1.96	1.08E-02
	tube development	584	54	27.79	1.94	2.78E-02
	regulation of developmental process	1511	128	71.91	1.78	1.01E-06
	regulation of cell population proliferation	972	78	46.26	1.69	4.37E-02
	anatomical structure morphogenesis	1527	112	72.68	1.54	3.13E-02

Analyses were performed using lists of significant (P<0.05) positive differentially expressed genes between ‘*STMN2* ^+^ alveolar fibroblasts’ and other alveolar fibroblasts, and between clusters of adventitial fibroblasts versus all other fibroblasts. ‘Gene set’ indicates the number of genes in the gene set, ‘Upregulated’ the number of genes from the gene set that are upregulated in the cluster, ‘Expected’ the number of genes from the gene set expected to be present if there is no enrichment.

Adventitial fibroblasts (overexpressing *COL14A1, GLI1* and *DCN*) (21, 22) were divided into four clusters, named after their most specific marker. ‘*CCL19* ^+^ adventitial fibroblasts’ overexpressed cytokine and chemokine genes (*CCL19, CCL7*), serum amyloid A1 (*SAA1*), fibroblast activation protein (*FAP*) and, compared with all other fibroblasts, GO analyses revealed significant enrichment in transcripts involved in inflammatory response and regulation of leukocyte proliferation (**Table 2**; **Supplementary Table 1**). ‘*CCN3* ^+^ adventitial fibroblasts’ overexpressed cellular communication network factor 3 (*CCN3*), matrillin 4 (*MATN4*) and also *FAP*, while GO analyses identified biological processes such as ‘regulation of cell migration’, ‘positive regulation of cell population proliferation’ and ‘regulation of developmental process’ as significantly overrepresented (**Table 2**; **Supplementary Table 1**). ‘*COL23A1* ^+^ adventitial fibroblasts’ overexpressed CXC motif chemokine ligand 14 (*CXCL14*), neurotrophic receptor tyrosine kinase 2 (*NTRK2*), signaling receptor and transporter of retinol (*STRA6*) as well as collagen type XXIII alpha 1 chain (*COL23A1*) and biological processes such as ‘positive regulation of cell migration’ and ‘animal organ morphogenesis’ were enriched in GO analyses (**Table 2**; **Supplementary Table 1**). The last cluster of adventitial fibroblasts, ‘*CCBE1* ^+^ adventitial fibroblasts’, overexpressing mediator complex subunit 13L (*MED13L*), hemicentin-1 (*HMCN1*) and collagen and calcium binding EGF domains 1 (*CCBE1*), also had an increased expression of transcripts associated with morphogenesis processes such as ‘circulatory system development’, ‘tube development’ and ‘regulation of developmental process’ (**Table 2**; **Supplementary Table 1**). In summary, lung fibroblasts exhibited heterogeneity in transcriptional profiles, possibly reflecting substantial functional diversity.

### 3.5 Lung smooth muscle cells divide into airway and vascular axis

**Figure 4:**
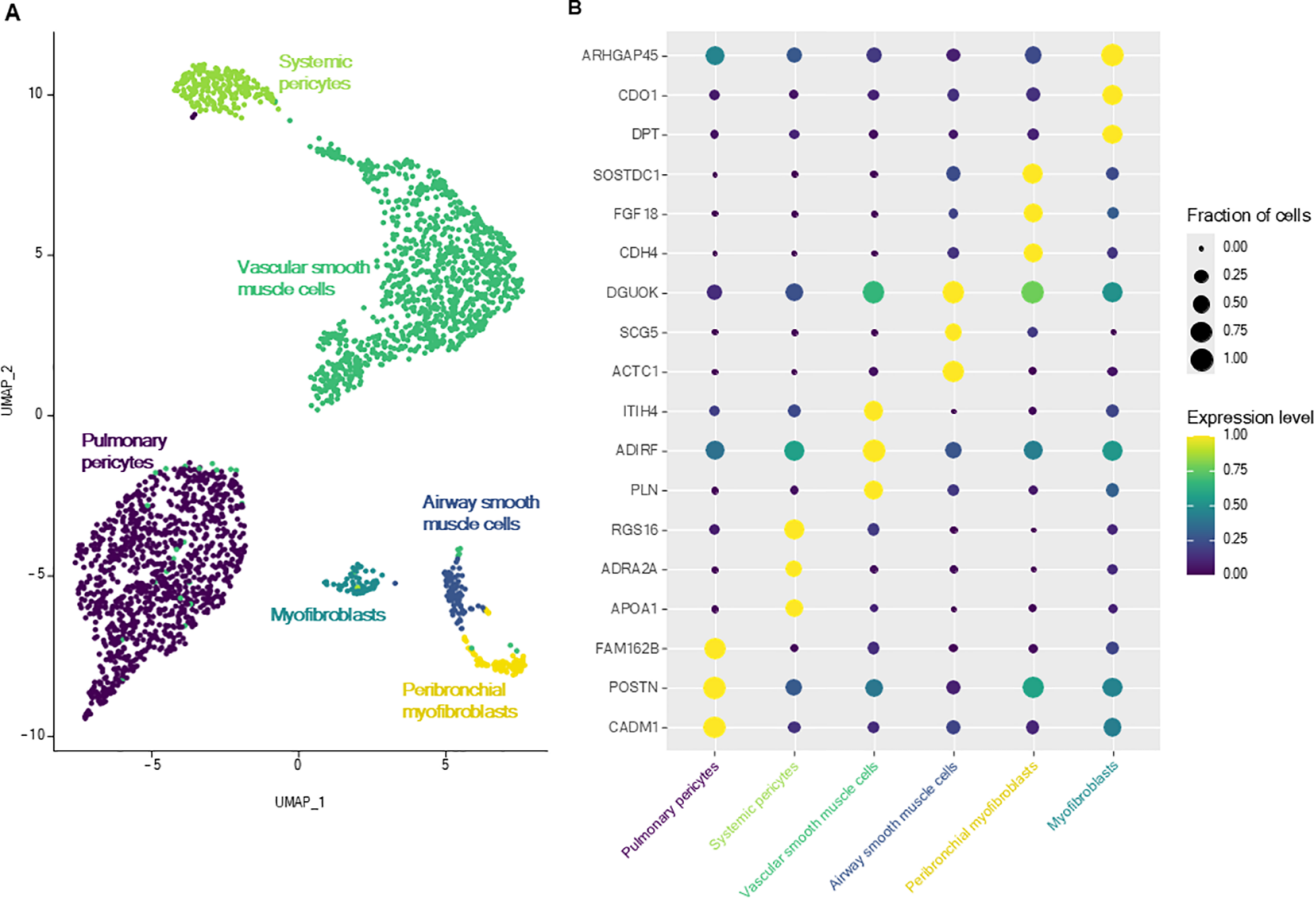
Lung smooth muscle cells. **(A)** UMAP representation of the six smooth muscle cell subtypes. **(B)** Dot plot representing the specific expression of muscle cell subtypes markers.

Smooth muscle cells were divided into six different cell subpopulations (**Figure 4**), each defined by specific DEGs compared with all other muscle cells (**Supplementary Table 3**). They were classified according to the two remaining axis of mesenchymal cells: airway and vascular axis (21). Airway axis was constituted, proximally to distally, by airway smooth muscle cells (overexpressing *ACTC1*) and by peribronchial myofibroblasts (overexpressing *SOSTDC1* and *FGF18*) (3, 21). Vascular axis (*NOTCH3* ^+^) was composed of, proximally to distally, vascular smooth muscle cells and two clusters of pericytes, which exhibited lower expression of contractility genes (e.g. *ACTA2*, *TAGLN*, *MYH11*) (2, 21, 22). DEGs of the largest (*POSTN*, *FAM162B*, *HIGD1B*) and of the smallest pericyte cluster (*APOA1*, *ADRA2A*, *RGS16*, *COL12A1*, *CLU*) were previously described as markers of pericytes from the pulmonary and systemic circulation, respectively (3). The remaining cluster expressed both markers of smooth muscle cells (*ACTA2*, *MYH11*) and fibroblasts (*DPT*, *ASPN*, collagens) and was thus annotated as ‘myofibroblasts’ (2, 20).

### 3.6 Lung immune cells are identified with high resolution

**Figure 5:**
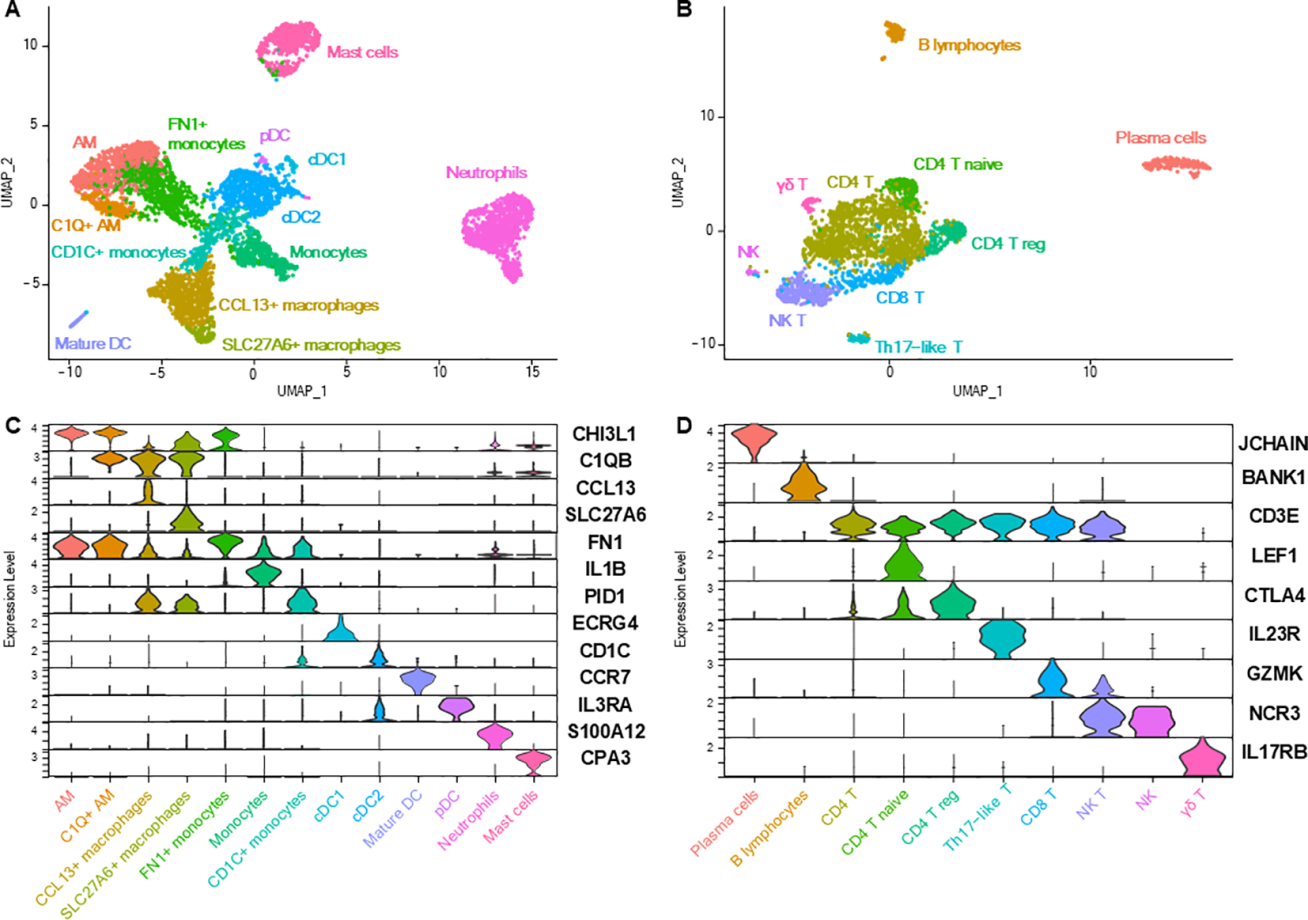
Lung immune cells. **(A)** UMAP representation of the 13 distinct myeloid cell clusters. **(B)** UMAP representation of the 10 distinct lymphoid cell subtypes. **(C, D)** Violin plots depicting the expression of key markers for each immune cell subtype.

Lung immune cells were divided into myeloid and lymphoid cells, which were individualized for re-clustering to increase resolution. Myeloid cells were constituted of 13 subpopulations (**Figures 5A, C**; **Supplementary Table 4**), including seven clusters of macrophages and monocytes, four clusters of dendritic cells (DC), in addition to neutrophils and mast cells.

Two clusters of alveolar macrophages (AM) were identified based on their overexpression of, among others, *MARCO*, *SIGLEC1* and *PPARG* (1, 3, 4, 23). The smaller AM cluster differed from the main cluster in its expression of genes of the complement component subunits (*C1QA, C1QB, C1QC*). Another macrophage cluster, named ‘*CCL13* ^+^ macrophages’ overexpressed complement genes, cytokines and chemokines such as *CCL13*, *CCL14* and *CX3CX1*, as well as *STAB1, F13A1* and *LYVE1*. GO analyses based on DEGs between *CCL13* ^+^ macrophages and AM (**Supplementary Table 5**) identified enriched biological processes such as ‘endocytosis’, ‘leukocyte chemotaxis’, ‘positive regulation of macromolecule metabolic process’ (**Table 3**; **Supplementary Table 1**). ‘*SLC27A6* ^+^ macrophages’ shared markers with ‘*CCL13* ^+^ macrophages’ while DEGs compared with the latter (*SLC27A6*, *GPNMB*, *CTSK*, *DHDH*, *APOE*; **Supplementary Table 5**) revealed through GO analysis an enrichment in ‘regulation of amyloid-beta clearance’, ‘positive regulation of endocytosis’, ‘negative regulation of protein metabolic process’ (**Table 3**; **Supplementary Table 1**). Macrophage clusters seemed to exhibit a degree of overlap between gene expression profiles, especially regarding the expression of *MRC1, C1QA, C1QB, C1QC*, as in humans, and as opposed to mice, in which the expression of those genes are restricted to one macrophage cluster (24).

**Table 3:** Gene ontology analysis of the transcriptomic profiles of specific monocytes or macrophages clusters.

Cluster	Biological process	Gene set	Upregulated	Expected	Fold enrichment	P-value
‘*CCL13* ^+^ macrophages’	leukocyte chemotaxis	85	14	2.69	5.2	2.90E-03
	endocytosis	348	36	11.02	3.27	3.43E-06
	leukocyte differentiation	282	27	8.93	3.02	2.46E-03
	regulation of cell activation	400	31	12.67	2.45	4.83E-02
	positive regulation of immune system process	678	50	21.47	2.33	2.37E-04
	positive regulation of macromolecule metabolic process	2026	100	64.15	1.56	4.72E-02
‘*SLC27A6* ^+^ macrophages’	regulation of amyloid-beta clearance	8	3	0.03	86.41	2.99E-02
	positive regulation of endocytosis	101	6	0.44	13.69	3.53E-02
	negative regulation of protein metabolic process	288	9	1.25	7.2	3.08E-02
‘*FN1* ^+^ monocytes’	antigen processing and presentation of exogenous peptide antigen via MHC class II	24	8	0.26	31.07	6.74E-07
	peptide antigen assembly with MHC protein complex	19	5	0.2	24.53	9.59E-03
	positive regulation of endocytosis	101	9	1.08	8.3	9.77E-03
	regulation of cell activation	400	16	4.29	3.73	4.97E-02
	protein catabolic process	638	23	6.85	3.36	2.91E-03
‘*CD1C* ^+^ monocytes’	peptide antigen assembly with MHC class II protein complex	15	5	0.18	28.53	3.91E-03
	antigen processing and presentation of exogenous peptide antigen via MHC class II	24	6	0.28	21.4	1.85E-03
	adaptive immune response	248	14	2.9	4.83	9.75E-03
	regulation of immune response	650	23	7.59	3.03	1.76E-02
	positive regulation of immune system process	678	23	7.92	2.9	3.53E-02

Analyses were performed using lists of significant (P<0.05) positive differentially expressed genes between ‘*CCL13* ^+^ macrophages’ and ‘Alveolar macrophages’, between ‘*SLC27A6* ^+^ macrophages’ and ‘*CCL13* ^+^ macrophages’, between ‘*FN1* ^+^ monocytes’ and ‘Monocytes’ and between ‘*CD1C* ^+^ monocytes’ and ‘Monocytes’. ‘Gene set’ indicates the number of genes in the gene set, ‘Upregulated’ the number of genes from the gene set that are upregulated in the cluster, ‘Expected’ the number of genes from the gene set expected to be present if there is no enrichment. MHC, major histocompatibility complex.

Monocytes overexpressed *IL1B*, *IL1A, EREG*, *VCAN* and *MAFB* (1, 25). ‘*FN1* ^+^ monocytes’, compared with other monocytes, overexpressed genes (such as *APOC1*, *C1QC, LMNA*, *HLA-DQB2*, *DLA-DRA*, *MRC1*; **Supplementary Table 5**) enriched in biological processes related to ‘antigen processing and presentation’, ‘regulation of endocytosis’ and ‘protein catabolic process’ (**Table 3**; **Supplementary Table 1**). ‘*CD1C* ^+^ monocytes’, compared with other monocytes, overexpressed genes (such as *PKIB, MRC1, HLA-DBQ2, DLA-DQA1, FCER1A*; **Supplementary Table 5**) enriched in biological processes related to ‘antigen processing and presentation’, ‘adaptive immune response’ and ‘regulation of immune response’ (**Table 3**; **Supplementary Table 1**) and might represent a transitional state towards DC or monocyte-derived DC.

Four different types of DC were identified based on their expression profiles: plasmacytoid DC, mature DC, myeloid/conventional DC 1 (cDC1), and myeloid/conventional DC 2 (cDC2) (1, 3, 18, 19, 23). Among notable genes, *IDO1*, an immunotherapy target expressed by mature DC, is also expressed by human DC, while being expressed at very low levels in mouse DC (24, 26). Neutrophils were recognizable thanks to their overexpression of *S100A12* or *SELL* (1, 18) and mast cells expressed very specific markers such as *KIT*, *CPA3* or *MS4A2* (1, 2, 20). No eosinophils nor basophils were identified.

Lymphoid cells were also characterized with a very high resolution, allowing the profiling of 10 cell clusters (**Figures 5B, D**; **Supplementary Table 6**). The expression of *CD3E* allowed the discrimination of T lymphocytes from other lymphoid cells (1, 2). Besides the main group of *CD4* ^+^ T cells (expressing *CD4*, *IL7R*, *THY1*), three additional *CD4* ^+^ T subpopulations could be further discriminated and classified as naïve T cells (expressing *CCR7*, *LEF1*, *SELL*), regulatory T cells (with higher expression of *CTLA4* and *TNFRSF4*) and Th17-like T cells (expressing genes of Th17-associated proteins *IL23R*, *IL17A, CCR6* and *RORA*) (2, 18, 27). *CD8A* ^+^ T cells comprised *CD8* ^+^ cytotoxic T cells (expressing cytokines and cytotoxicity-associated markers such as granzyme and killer cell lectin-like receptor genes) and natural killer (NK) T cells (expressing *NCR3* in addition to other genes of proteins associated with cytotoxicity) (2, 18, 27). A last cluster of T cells was identified as ‘γδ T cells’ (overexpressing *IL17RB* and *GATA3*) (18). A small cluster of NK cells (negative for *CD3E* but expressing *NFKBID*, *NCR3*, *KLRK1*, *CD96*) was also identified (18, 20, 28). Forming two distant clusters, B lymphocytes (*FCRLA* ^+^) and plasma cells (*JCHAIN* ^+^) were present as well (1–3, 20).

### 3.7 Epithelial and endothelial cells markers cluster according to canonical cell types

**Figure 6:**
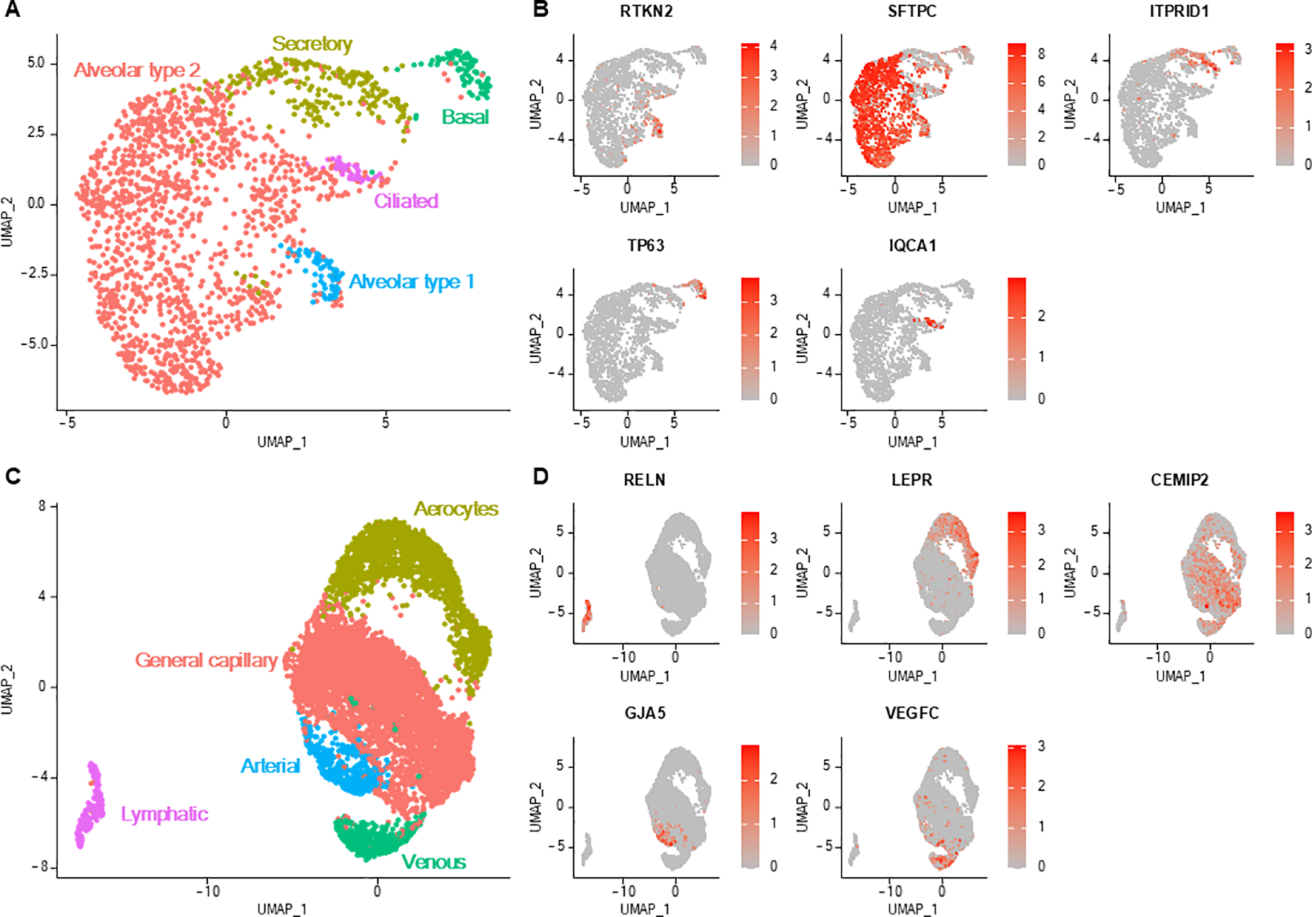
Lung epithelial and endothelial cells. **(A)** UMAP representation of the five distinct epithelial cell subtypes. **(B)** Feature plots depicting the normalized expression level of specific markers for each epithelial cell type. Color scales represent the expression level of each gene. **(C)** UMAP representation of the five distinct endothelial cell subtypes. **(D)** Feature plots depicting the normalized expression level (red color scale) of specific markers for each endothelial cell type.

Lung epithelial cells clustered into five different cell types, and each exhibited their own transcriptional profile (**Figures 6A, B**; **Supplementary Table 7**). Conservatively to human data (2, 3, 20), canine lung epithelial cells spread into alveolar type 2 cells (overexpressing genes of surfactant proteins and napsin A protein, the latter being used as a lung carcinoma marker in dogs), alveolar type 1 cells (expressing *AGER*), secretory cells (expressing *SCGB1A1*, *MUC5B*), basal cells (expressing *KRT14* and transcription factor *TP63*) and ciliated cells (expressing *CAPS*, *FOXJ1*, *CCDC78*, *HYDIN*). The expression levels of growth factor receptor genes (*EGFR* and *ERBB2*) and proliferation marker genes (*PCNA* and *MKI67*) in epithelial cells were not significantly higher in unaffected lung samples adjacent to a focal tumor compared with samples originating from healthy lungs (**Supplementary Figure 1**). Moreover, enrichment analysis did neither reveal cancer-associated genes (**Supplementary Table 8**) nor enrichment in cancer-associated biological processes (**Supplementary Table 1**) in epithelial cells from unaffected tumor-adjacent lung samples as compared to those from lung samples of dogs exempt of lung disease.

Lung endothelial cells were distributed into five cell clusters (**Figures 6C, D**; **Supplementary Table 9**). Lymphatic endothelial cells were distinguished from vascular endothelial cells with their low expression of *VWF* (a vascular endothelial cell marker) and their overexpression of specific markers (including *PDPN* and *PROX1*) (2, 29). Vascular endothelial cells were divided into endothelial cells constituting capillaries of the pulmonary circulation, namely aerocytes (also exhibiting low *VWF* expression), capillaries of the general circulation (general capillary endothelial cells), arteries (with comparatively higher *GJA5* and *BMX* expression) and veins (higher *ACKR1* expression) (2, 29).

### 3.8 Summary of canine lung cell transcriptional diversity

By combining all cell compartments, a total of 46 different cell clusters were identified, constituting an extensive atlas of canine lung cells. A summary of transcriptional signatures of each of the 46 cell clusters is provided in **Table 4** and complete lists of DEGs of one cell cluster versus all other lung cells are reported in **Supplementary Table 10**. The contribution of each lung sample to every cell cluster is documented as raw counts (**Supplementary Table 11**) and percentages (**Supplementary Figure 2**). Overall, all four samples contributed to nearly every lung cell cluster, enhancing the cellular diversity of the single-cell atlas. Notable exceptions were mast cells, with 87 percent originating from Lung 1 despite being present in all samples; Schwann cells, with 94 percent derived from Lung 3 and none detected in Lung 4 (which provided the lowest total number of cells), and ciliated cells, which were absent from Lung 2.

**Table 4:** Summary of the transcriptional signatures of canine lung cells.

Cell type	Markers
Mesenchymal cells	
Schwann cells	*NRXN1, CDH19, SCN7A, SNCA, NTNG1, NRN1, MPZ*
Muscle cells	*ACTA2, TAGLN, MYH11*
Pericytes (pulmonary circulation)	*CADM1, POSTN, FAM162B, F3, SLC4A4, ENPP2, HIGD1B, PAG1*
Pericytes (systemic circulation)	*APOA1, ADRA2A, PDE1A, COL12A1, IL33, ADGRF5, RGS16*
Vascular smooth muscle cells	*PLN, ADIRF, DSTN, ITIH4, FAM13C, DGUOK, MAP1B, FABP3*
Airway smooth muscle cells	*ACTC1, SCG5, SEMA3C, NWD2, CACNA2D3, MT3, TENM1*
Peribronchial myofibroblasts	*CDH4, CCBE1, FGF18, MFAP5, SOSTDC1, ADAMTS6, EPHA7, SMOC2*
Myofibroblasts	*NKAIN3, CADM2, COL6A5, C3, PTPRD, DPT, ADRA1A, GPM6B*
Fibroblasts	*COL1A1*
Alveolar fibroblasts	*NAA16, MACF1, SPECC1L, IFT27, VEGFD, NPNT, ASPN*
*STMN2* ^+^ alveolar fibroblasts	*STMN2, PRG4, IL33, COL6A6, CCL7, CCBE1, S100A1*
*CCBE1* ^+^ adventitial fibroblasts	*MED13L, HMCN1, SETBP1, CCBE1, ZFPM2, COL6A6, XYLT1*
*CCL19* ^+^ adventitial fibroblasts	*PI3, CCL19, CCL7, FAP, F3, NFKBIA, SAA1, CXCL12*
*CCN3* ^+^ adventitial fibroblasts	*PI3, CCN3, RIT2, MATN4, PCOLCE2, SMOC2, GPC6, RBP4*
*COL23A1* ^+^ adventitial fibroblasts	*AQP1, NRTK2, LAMA2, LSP1, STRA6, SNF385D, COL23A1, PLCL1*
Immune cells	*PTPRC*
Myeloid immune cells	
Alveolar macrophages (AM)	*CHI3L1, CPNE6, CLU, GDE1, CDC42EP3, BPI, MARCO, PPARG*
*C1Q^+^* AM	*C1QB, C1QC, C1QA, RDH16, CHI3L1, CPNE6, MARCO, PPARG*
*CCL13* ^+^ macrophages	*CCL13, C1QC, STAB1, PID1, F13A1, PLTP, CCL14, CCL8, C1QA*
*SLC27A6* ^+^ macrophages	*SLC27A6, PLTP, TREM2, MMP2, CTSK, STAB1, DHDH, GPNMB*
Monocytes	*IL1B, EREG, IL37, SNAI1, VCAN, SERPINB2, IL1A, CCL3, MAFB*
*FN1* ^+^ monocytes	*FN1, SMPDL3A, LMNA, RBP4, GLDN, IL1A, MAFB, APOC1*
*CD1C* ^+^ monocytes	*PID1, IL1RN, IL1R1, ATF3, MMP12, LYZ, MAFB, CD1C*
Myeloid/conventional DC 1	*CLNK, ECRG4, CLEC1B, HOOK1, DOCK5, DLA-DOA, CADM1, IRF8*
Myeloid/conventional DC 2	*PKIB, NAPSA, NCAM2, NR4A2, DLA-DOA, TRABD2A, PPM1J, CD1C*
Mature DC	*CCR7, SLC22A23, SLCO5A1, IDO1, PLEKHG1, FSCN1, IL4I1, CD40*
Plasmacytoid dendritic cells (DC)	*GPHA2, SHANK2, IL3RA, SPATA6, IGKC, HMGCS1, PPM1J, TCF4*
Neutrophils	*S100A12, S100A9(ENSCAFG00000029470), IL18BP, SAT1, CXCL8, SOD2, CD4, SELL*
Mast cells	*CPA3, MS4A2, MAGI2, CMA1, KIT, SYTL3, FCER1A, HPGDS*
Lymphoid immune cells	
CD4 T lymphocytes	*JAZF1, IL7R, S100A8, LGALS3*, *INPP4B, ICOS, S100A5, CD3E*
CD4 naïve T cells	*CCR7, LEF1, CTPS1, RGS10, SELL*, *IGF1R, TLE1, USP12*
CD4 regulatory T cells	*CTLA4, DBX2, TNFRSF4, TNFRSF18, IKZF2, LGALS3, CD28*
Th17-like T cells	*IL23R, BLK, IL1R1, CPNE8*, *SYNDIG1, IL1R2, IL17A, CCR6, RORA*
CD8 cytotoxic T cells	*CCL5, CCL4, GZMK, CCL3, KLRK1, CTSW, GZMB, CD8A*
Natural killer T cells	*NCR3, KLRD1, GZMB, KLRB1, GZMA, TXK, KLRK1, FASLG*
Natural killer cells	*SNCG, F2RL3, KLRB1, FCER1G, KLRK1, CRTAM, NCR3, CD96*
γδ T cells	*PTGES, PDE7B, PDE11A, IL17RB, CRLF2, IL1RL1, SLC4A4, GATA3*
B lymphocytes	*ARHGAP24, BANK1, TNFRSF13C, BCL11A, DLA-DOA, FCRLA, MS4A1, CCR7*
Plasma cells	*JCHAIN, MZB1, POU2AF1, TNFRSF17, DERL3, TXNDC5, FKBP11, RARRES2*
Epithelial cells	*EPCAM*
Alveolar type 1 cells	*RTKN2, ZNF365, SEMA6D, CAV2, CAV1, TIMP3, AGER*
Alveolar type 2 cells	*SFTPC, NAPSA, C5, LRRK2, SLC34A2, ACOXL, SFTPB, SFTPD*
Secretory cells	*ITPRID1, KCNIP4, SCGB1A1, AQP5, GPX2, CHL1, CLEC10A, NAV3*
Basal cells	*TP63, IL33, CNTNAP5, RNASE4, GABRE, CLDN1, SEMA5A, COL21A1*
Ciliated cells	*IQCA1, DCDC1, DNAH5, RIBC2, ROPN1L, CFAP126, TNNI3, MORN5*
Endothelial cells	*PECAM1*
Lymphatic endothelial cells	*RELN, NRP2, KCTD12, TSHZ2, MRC1, PROX1, LSP1, TBX1, PDPN*
Aerocytes	*LEPR, CFI, PLXNC1*, *CNTNAP2, KDR, EMP2, RGS6, EDNRB*
General capillary endothelial cells	*LYVE1, SPARCL1, CEMIP2, PTPRB, CADM1, CD36*
Arterial endothelial cells	*PDE3A, GJA5, BMX, BMPER, CLU, MECOM, MGP, LTBP4*
Venous endothelial cells	*VEGFC, ADGRG6, RNF144B, SELP, TIMP1, ACKR3, VCAM1, ACKR1*

### 3.9 Human lung homology analysis

The final annotated dataset combining all canine lung cell types was integrated with a single-cell reference atlas of the healthy human lung (4). Hierarchical clustering allowed the evaluation of cell type homologies between species. Even considering the differences in the level of final cell type annotations, canine lung cells showed a high degree of homology to human lung cells within each tissue compartment (**Supplementary Figure 3**). All endothelial cell types, and almost all epithelial cell types, except for basal cells, paired off 1:1 in terminal clades, suggesting a high degree of similarity. Among immune cells, canine and human mast cells, B lymphocytes, CD4 T cells, CD8 T cells, plasma cells DC1, DC2, mature (migratory) DC, alveolar macrophages also paired off 1:1 in terminal clades. Within mesenchymal cells, canine and human alveolar fibroblasts clustered together, and human adventitial fibroblasts clustered on the same clade as all four canine adventitial fibroblasts clusters. We also identified subtle differences between species. For example, canine *CD1C* ^+^ monocytes clustered with human DC2, which would strengthen the hypothesis that this cluster might represent a transitional state towards DC. While canine pericytes from the pulmonary circulation paired perfectly with human pericytes, canine pericytes from the systemic circulation seemed more similar to other canine and human smooth muscle cells. Additionally, canine ‘*STMN2* ^+^ alveolar fibroblasts’ paired 1:1 with human peribronchial fibroblasts. Taken together, the cross-species analysis underscores the similarities in lung cell transcriptional profiles, while also drawing attention to potential differences between the two species.

## 4 Discussion

This scRNA-seq atlas of healthy canine lung identified 46 transcriptionally distinct cell clusters and provided the molecular signatures for each of them, increasing considerably our knowledge of canine lung cellular biology and diversity. Our analysis revealed six distinct fibroblasts clusters. Such heterogeneity may reflect a diversity of fibroblasts activation states and possibly various functions, as some fibroblast clusters seem involved in immune regulatory functions. Regarding immune cells, the high resolution of the present analysis allowed the identification of rarer cell types such as γδ T cells, unconventional T cells that were already described in canine peripheral blood by scRNA-seq (18, 27). Additionally, lung smooth muscle cells, epithelial cells and endothelial cells were relatively easily identified using classification systems and markers described in humans (2–4, 29). Finally, homology analysis between canine and human lungs showed a high degree of similarity in lung cell transcriptional profiles while also highlighting potential differences.

In the literature, there is no description of single cell expression profiles of fibroblasts subsets in dogs. Indeed, in existing studies, fibroblasts are either absent, e.g. in BALF (1, 5), or are presented as a one entity (19, 30, 31). After integration, canine alveolar and adventitial fibroblasts from our dataset mapped with human alveolar and adventitial fibroblasts, respectively, except for the cluster of ‘*STMN2* ^+^ alveolar fibroblasts’ mapping with human and canine adventitial fibroblasts, which might suggest some continuum in fibroblasts transcriptional profiles. Interestingly, we could find particular resemblances between some fibroblast clusters present in our datasets and fibroblast subpopulations newly described in the healthy human lung (3). ‘*CCL19* ^+^ adventitial fibroblasts’ shared markers (such as *CCL19* and *CXCL12*) with a fibroblast subset likely exerting immune-recruiting properties and mapped to rare immune infiltrates in the bronchus (3). Interestingly, ‘*CCL19* ^+^ adventitial fibroblasts’ and ‘*CCN3* ^+^ adventitial fibroblasts’ overexpressed fibroblast activation protein *(FAP*), a marker of activated fibroblasts and cancer-associated fibroblasts, including in canine idiopathic pulmonary fibrosis and canine lung cancer (32). ‘*COL23A1* ^+^ adventitial fibroblasts’ shared key markers (*COL15A1*, *ENTPD1*, *PLCL1*) with peribronchial fibroblasts, a subpopulation specifically localized around the airway epithelium, which is enriched in idiopathic pulmonary fibrosis and may be implicated as a key cell type in lung disease (3). Regarding other mesenchymal cells, the cluster of lung pericytes from the systemic circulation shares a dozen specific markers (including *APOA1, ADRA2A, RGS16* and *ADAMTS4*) with a cluster of *APOA1* ^+^ smooth muscle cells described in canine arteries (31).

Although scRNA-seq studies conducted so far on different canine samples (1, 5, 18, 19, 27, 30) have provided valuable help, the classification of lung immune cells, especially monocyte and macrophages, remained challenging. Indeed, conventional markers arising from human and mouse studies are sometimes unhelpful for cell identification in dogs, due to incomplete annotation of the canine genome, species differences regarding transcriptome, or occasional low transcript abundance (18). For example, *CD14* and *CD16*, often used to characterize monocyte populations, lack annotation in the reference transcriptome used in this study (CanFam3.1). In this study, alveolar macrophages shared their most statistically significant makers (including embryonic-derived AM marker *MARCO*) with the cluster of alveolar macrophages mainly populating healthy canine BALF (1). *FN1* ^+^ monocytes shared top markers with a cluster of *MARCO^-^FN1^+^* macrophages from canine BALF, enriched in cytokine genes, which was considered as monocyte-derived macrophages or monocytes (1). In canine lungs, *FN1* ^+^ monocytes and ‘*SLC47A6* ^+^ macrophages both expressed *SPP1*, which is a marker of monocyte-derived macrophages in the integrated human lung cell atlas (4). Interestingly, *FN1^+^SPP1^+^* monocyte-derived macrophages are believed to be involved in the pathogenesis of canine idiopathic pulmonary fibrosis (5, 33) and profibrotic *SPP1* ^+^ monocyte-derived macrophages were also reported in human COVID-19, pulmonary fibrosis and lung cancer (4). Using the overexpression of a combination of human (4, 23) and mouse (25, 34–36) markers (*CX3CR1, F13A1, STAB1, LYVE1, C1QA, C1QC, C1QB*), *CCL13* ^+^ macrophages may be classified as interstitial macrophages. Furthermore, this cluster did not match any cluster present in canine BALF, which did not contain any interstitial macrophages (1, 5). *CCL13* ^+^ macrophages shared markers (*STAB1, C1QA, C1QC, C1QB, CCL7*) with macrophages identified in canine duodenum (30), another tissue containing postnatal-derived macrophages which have a phenotype similar to interstitial macrophages in mice (34). Additionally, the gene expression profiles of immune cells did not allow us to discriminate lung-resident from intravascular immune cells using lung immune cell residency signatures as reported in humans (2), possibly because resident and circulating cells clustered together and were not distinguishable, or because we lack appropriate discriminating markers in dogs. Additional studies with a spatial component would be highly valuable to confirm the localization of cell subpopulations in the tissue, particularly when performing comparisons with diseased tissues, to better understand the nature and localization of cells implicated in the disease pathophysiology.

This study has some limitations that warrant consideration. As only four dogs were used for this study, all cell populations from healthy canine lungs may not be fully represented. Since the cell type proportions differed between individuals, the transcriptome of some cell types may be driven primarily by one sample (e.g.: mast cells in Lung 1), suggesting that increasing the number of healthy lung samples could provide a more accurate and comprehensive representation of all lung cell transcriptomes. However, the study included dogs that were middle to old adults, representing three different breeds and sizes to approximate the diversity of healthy canine lung cell populations.

Moreover, the tissue dissociation process, an essential step in scRNA-seq, is another factor that may influence the relative proportions of cell types. While dissociation must be efficient enough to release hard-to-dissociate cells, proteolytic digestion at 37°C can be harsh on more sensitive cell types (37). This stress can lead to changes in gene expression, such as the upregulation of heat shock proteins, or the depletion of fragile cell populations, including epithelial cells, which may consequently be underrepresented in the final data (37). Hence the relative abundance of each cell type should be interpreted with caution.

Furthermore, one strength of scRNA-seq is its ability to define the gene expression profiles of diverse cell populations within a sample (2). However, certain cell types may remain uncaptured due to their extreme rarity or the need for specialized isolation methods (2). For instance, as in similar studies with lung tissue, eosinophils were absent from our dataset, likely due to their high RNase content causing rapid mRNA degradation (1–3). Unexpectedly, mesothelial cells were not identified in our dataset due to the absence of expression of known mesothelial cell markers (*MSLN, CALB2, UPK3B, KLK11, ITLN1*). Although mesothelial cells are expected in peripheral lung samples, they still constitute a very rare cell type in single cell data, representing only 0.07 percent of all lung cells in the HCLA (4).

Additionally, our findings would greatly benefit from subsequent spatial validation. While scRNA-seq provides valuable transcriptional insights, it lacks spatial context, which is essential for understanding the differentiation, localization and functional roles of these cell types within the lung microenvironment. *In situ* validation – whether through smFISH for RNA detection or, even more critically, protein-based approaches such as immunostaining – would offer a more comprehensive and robust picture of these subsets. Given that mRNA expression does not always correlate with protein abundance, protein-level validation would be particularly valuable. Such spatial analyses would not only refine our understanding of these clusters but also strengthen the biological interpretations of their roles and interactions within the tissue.

Lastly, because obtaining fresh, healthy lung biopsies from dogs euthanized for unrelated reasons can be challenging in a clinical setting, we included biopsies from healthy lung tissue adjacent to primary lung tumors. This approach enabled us to expand our dataset and include diverse samples, but it also warrants cautious interpretation. The presence of cancer cells in tumor-adjacent healthy lung tissues was considered unlikely based on histopathology, the lack of expression of *EGFR* and *ERBB2*, expressed in respectively 73 and 69 percent of canine primary lung cancers (38), the lack of expression of proliferation markers used to identify replicating cells, and the analysis of differentially expressed genes and of biological process enrichments. However, only a limited marker panel was used, the molecular profiles of the primary tumors are unknown, and statistical power was limited due to the small sample sizes. Additionally, field effects from nearby tumors may have influenced gene expression in adjacent healthy tissue, although healthy lung tissues were collected with a margin of at least 2 cm from the visible tumor edge, which would further minimize the risk of contamination by tumor-associated effects.

In conclusion, this study provides a comprehensive molecular cell atlas of the canine healthy lung by describing 46 transcriptionally distinct lung cell clusters along with their gene expression signatures. Such atlas will provide the molecular foundation for investigating lung cell identities, functions and interactions in canine lung diseases. Additionally, the numerous similarities observed between canine and human lung cells highlighted the potential of the canine model to provide insights into human lung diseases.

## Data Availability

Raw and processed sequencing data can be found in ArrayExpress online repository (https://www.ebi.ac.uk/arrayexpress/) under the accession number E-MTAB-14296. The complete analysis code is publicly available at https://github.com/elodierizzoli/canine_lung_healthy. Any additional data requests can be made by contacting the corresponding author.
