# Supplementary material for: A single-cell RNA sequencing atlas of the healthy canine lung: a foundation for comparative studies
Source: Front Immunol. 2025 Mar 6;16:1501603. doi: 10.3389/fimmu.2025.1501603 (PMC11922831; doi:10.3389/fimmu.2025.1501603)
Supplement: Supplementary file 1 [file DataSheet1.docx]

Supplementary Material

**A single cell RNA sequencing atlas of the healthy canine lung:**

**a foundation for comparative studies**

**Elodie Rizzoli^1*^, Laurence Fievez^2,3^, Aline Fastrès^1^, Elodie Roels^1^, Thomas Marichal^2,4,5^, Cécile Clercx^1^**

^1^Department of Companion Animals Clinical Sciences, Faculty of Veterinary Medicine, University of Liège, Liège, Belgium

^2^Department of Functional Sciences, Faculty of Veterinary Medicine, University of Liège, Liège, Belgium

^3^Laboratory of Cellular and Molecular Immunology, GIGA Institute, University of Liège, Liège, Belgium

^4^Laboratory of Immunophysiology, GIGA Institute, University of Liège, Liège, Belgium

^5^Walloon Excellence in Life Sciences and Biotechnology (WELBIO) Department, WEL Research Institute, Wavre, Belgium

*** Correspondence:**Corresponding Author
elodie.rizzoli@uliege.be

# Supplementary tables

- Supplementary Table 1 – Gene Ontology analyses outputs.
- Supplementary Table 2 – Complete lists of differentially expressed genes between each fibroblast cluster and all other fibroblasts.
- Supplementary Table 3 – Complete lists of differentially expressed genes between each muscle cell cluster and all other muscle cells.
- Supplementary Table 4 – Complete lists of differentially expressed genes between each myeloid cell cluster and all other myeloid cells.
- Supplementary Table 5 – Complete lists of differentially expressed genes between specific myeloid cell clusters.
- Supplementary Table 6 - Complete lists of differentially expressed genes between each lymphoid cell cluster and all other lymphoid cells.
- Supplementary Table 7 – Complete lists of differentially expressed genes between each epithelial cell cluster and all other epithelial cells.
- Supplementary Table 8 – List of differentially expressed genes between epithelial cells originating from lung tissue adjacent to a focal tumor and epithelial cells originating from healthy lungs.
- Supplementary Table 9 - Complete lists of differentially expressed genes between each endothelial cell cluster and all other endothelial cells.
- Supplementary Table 10 – Complete lists of differentially expressed genes between each cell cluster and all other lung cells generated using FindMarkers() function.
- Supplementary Table 11 – Cell distribution across cell clusters and lung samples.

# Supplementary Figures


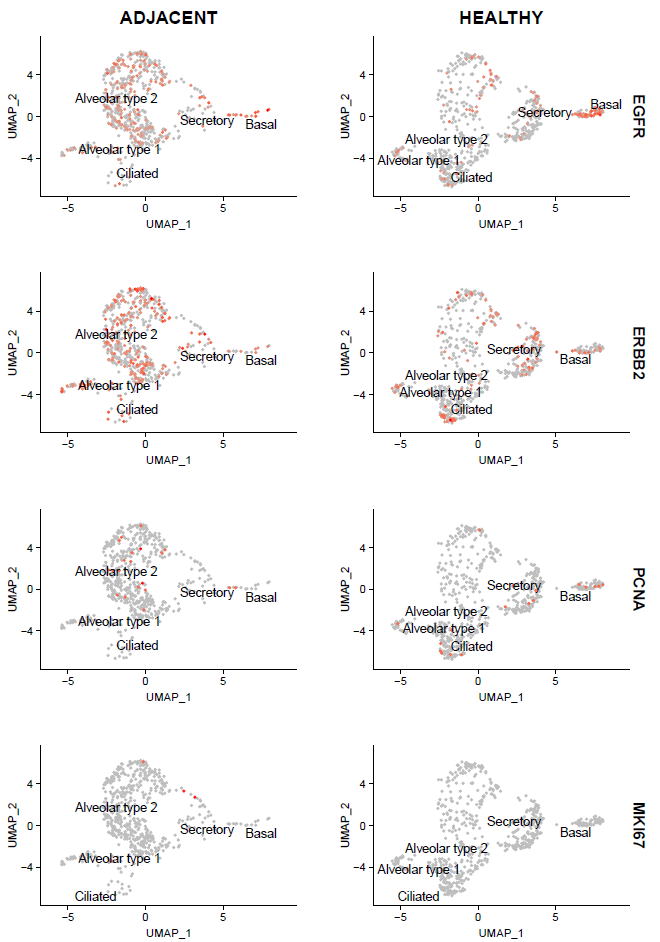


**Supplementary Figure 1.** Comparison of the expression of lung cancer-related genes between samples originating from unaffected lung tissue adjacent to a focal tumor (‘adjacent’) and samples originating from dogs exempt from lung disease (‘healthy’). The level of expression of growth factor receptors genes (*EGFR* and *ERBB2* coding for HER2) and proliferation marker genes (*PCNA* and *MKI67*) did not seem to be significantly different between ‘adjacent’ and ‘healthy’ samples.


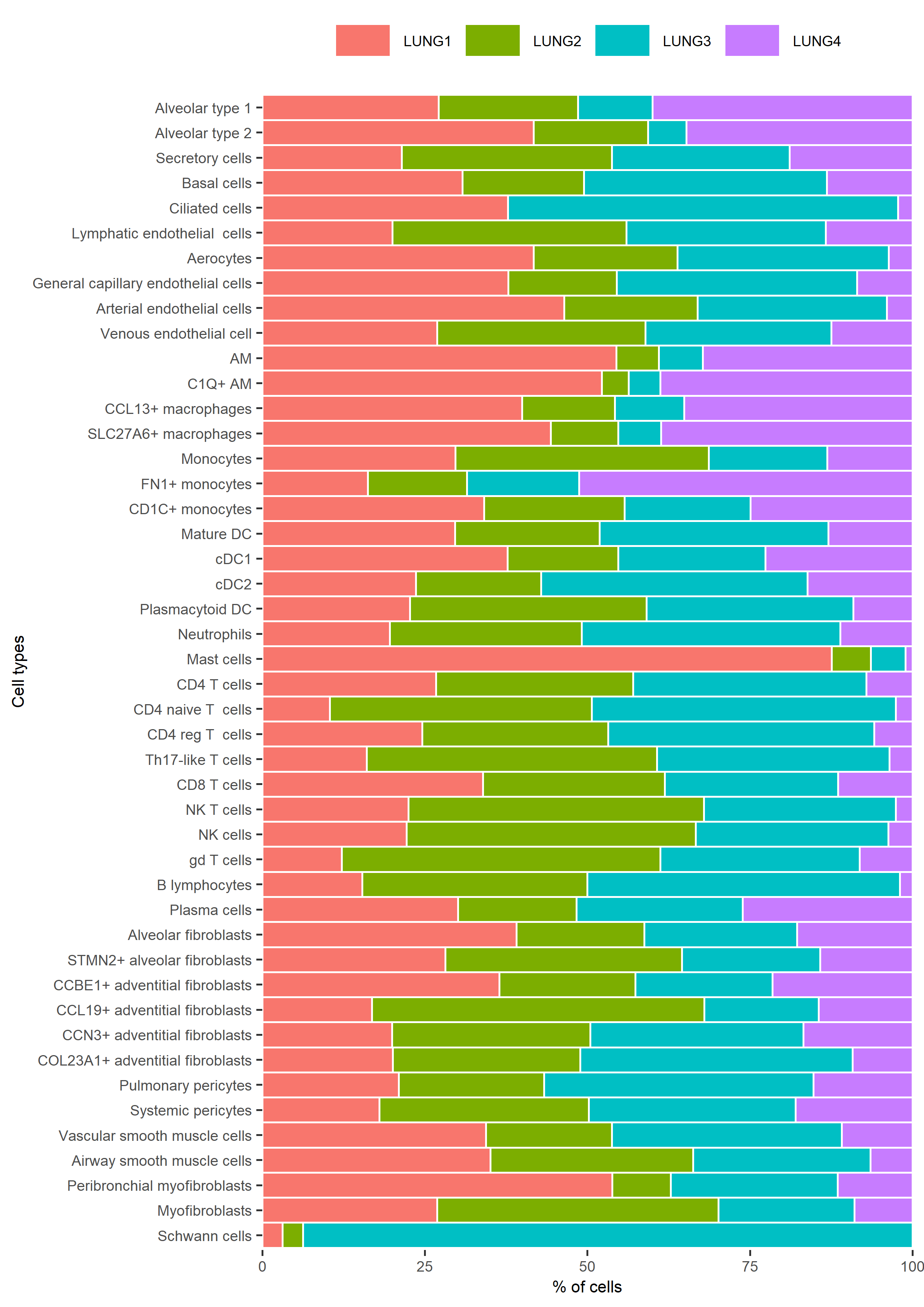


**Supplementary Figure 2.** Bar plot illustrating the proportion of each cell subtype provided by each lung sample. AM: alveolar macrophages; DC: dendritic cells; NK: natural killer, gd: gamma-delta.


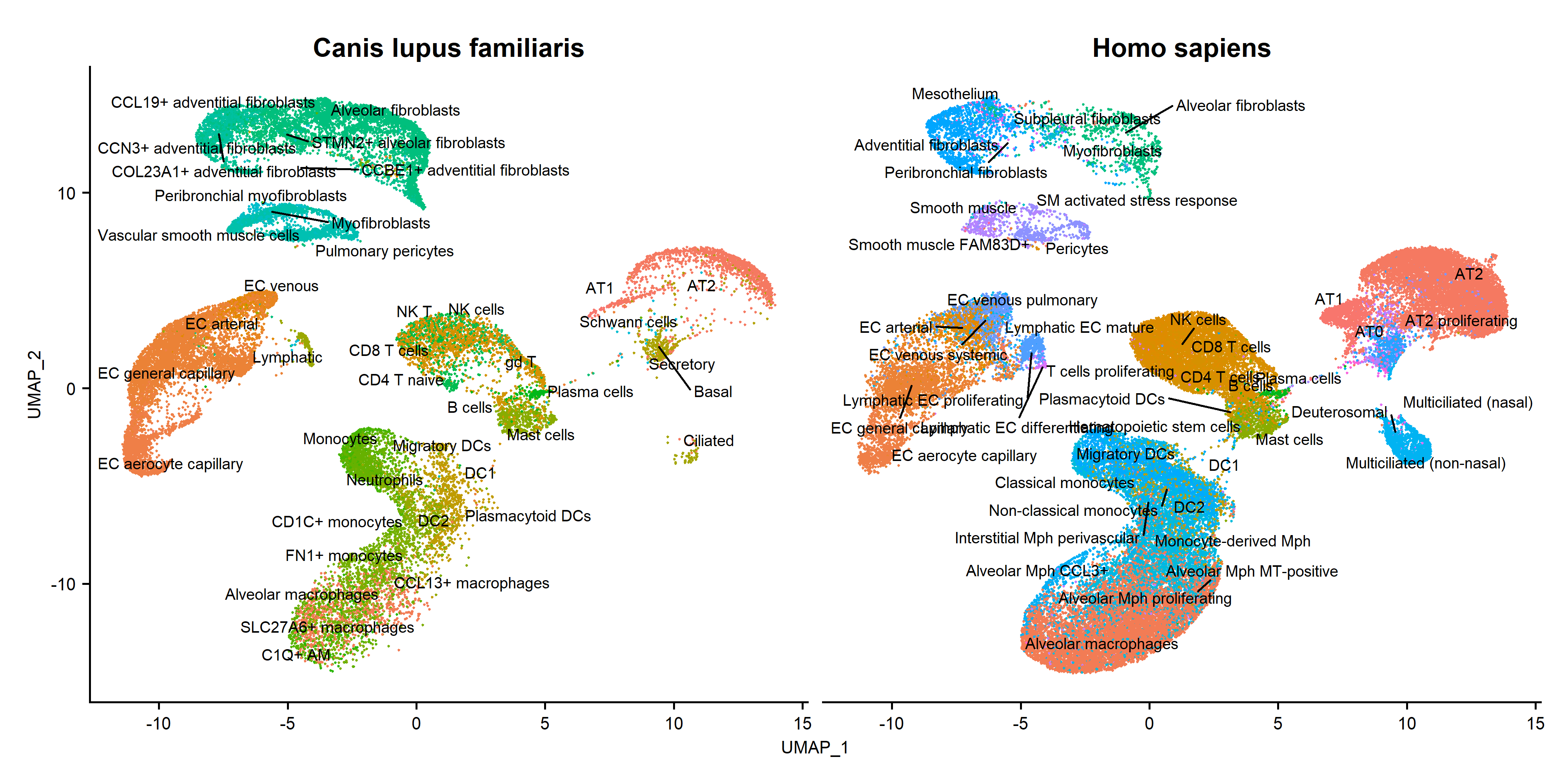


**B**

**A**


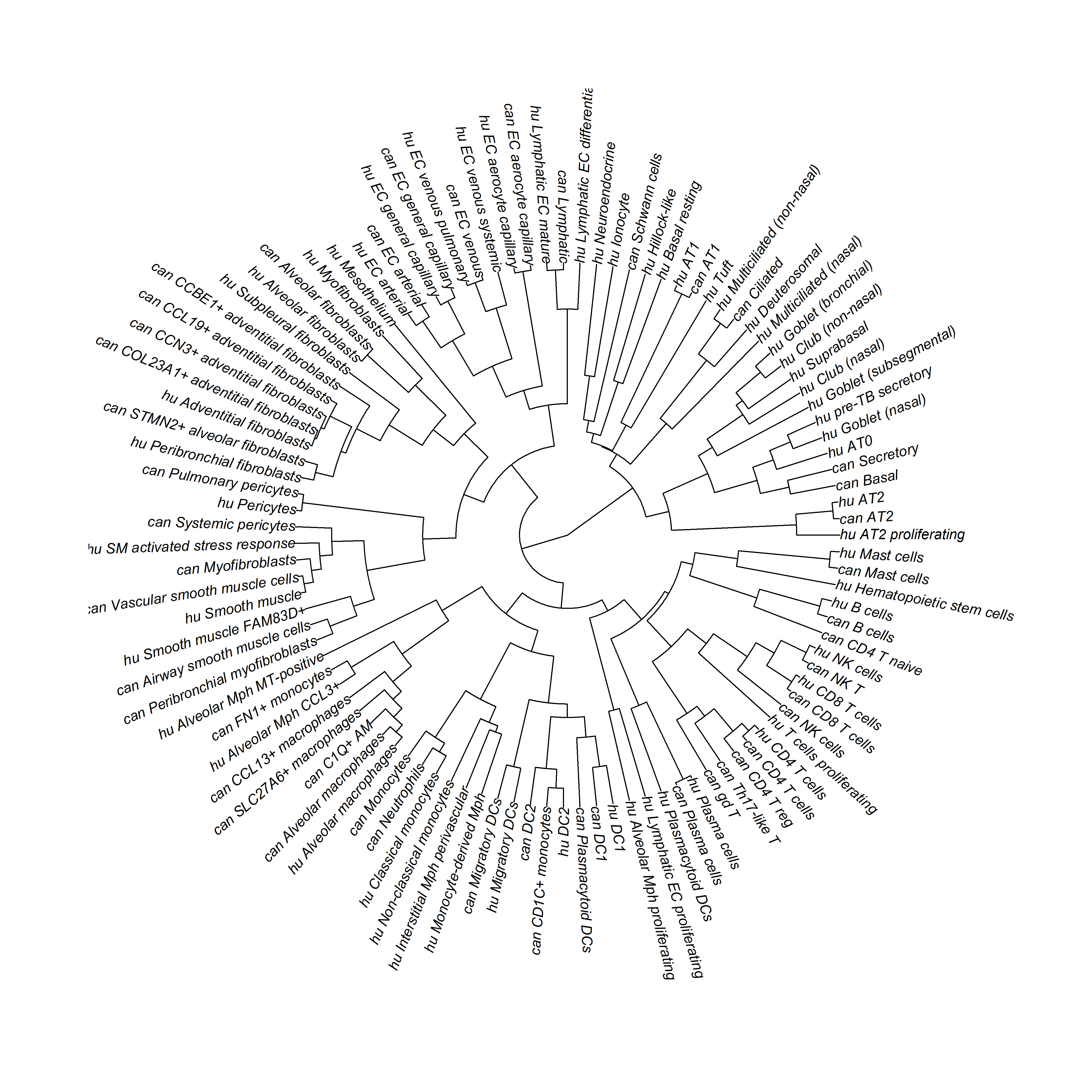


**Supplementary Figure 3.** Comparison of canine and human healthy lung cells. (A) UMAP depicting the final annotations of the integrated canine (left) and human (right) datasets. (B) Hierarchical clustering of the canine and human cell subtypes. Prefix “hu_” = human, “can_” = canine cell type.
